# Seronegative Disseminated Cryptococcosis: A Case Report

**DOI:** 10.1155/carm/5564622

**Published:** 2025-04-19

**Authors:** Maria Roell, Kevin Baker

**Affiliations:** Department of Internal Medicine, Medical University of South Carolina, Charleston, South Carolina, USA

## Abstract

Cryptococcal infection is a major cause of morbidity and mortality in immunocompromised patients, especially those with HIV/AIDS. The cryptococcal antigen (CrAg) lateral flow assay (LFA) has become an essential diagnostic tool due to its high sensitivity, specificity, and ability to produce rapid results. However, this test is not without limitations. This case details a patient with disseminated cryptococcosis with a negative serum CrAg LFA and an unremarkable brain MRI to demonstrate the importance of cerebrospinal fluid testing in high-risk immunocompromised individuals.

## 1. Background

Cryptococcal infection is a major cause of morbidity and mortality among immunocompromised patients. Despite the availability of antiretroviral therapy in the Western world, individuals with advanced HIV or AIDS remain at high risk for *Cryptococcus neoformans* infection. As the most common life-threatening mycosis in HIV patients, early and accurate diagnosis is crucial [1].

The cryptococcal antigen (CrAg) lateral flow assay (LFA) has become the gold standard for diagnosing cryptococcal disease due to its nearly 100% sensitivity and specificity and its ability to be performed at the bedside as a point-of-care test [2]. CrAg can be detected in serum, plasma, whole blood, and cerebrospinal fluid (CSF). Notably, a positive serum CrAg result has been found to precede the onset of cryptococcal symptoms by an average of 22 days [3]. False negative CrAg LFAs are rare but have occurred when excessive antigen relative to antibodies causes interference, known as the prozone effect [4].

The World Health Organization and many national HIV guidelines recommend routine serum CrAg screening for HIV-infected individuals with CD4 counts ≤ 100 cells/mm^3^. This proactive approach enables early detection of cryptococcal disease before symptoms appear [5]. The high diagnostic accuracy of this test has reduced reliance on CSF cultures, which can take several days to yield results and may delay diagnosis and treatment. The CrAg LFA provides results within the same day, facilitating timely identification and intervention [6].

In this report, we present a case of disseminated cryptococcal infection in a patient who had a negative serum CrAg LFA and unremarkable brain MRI 8 days prior to the onset of disseminated *Cryptococcus* symptoms. This case highlights the importance of performing CSF testing in immunocompromised patients when there is suspicion for fungal infection.

## 2. Case Report

A 35-year-old Hispanic female with no prior medical history presented to the emergency department with several days of right-sided abdominal pain and sudden, severe headaches. On examination, she was afebrile but tachycardic with notable rigors and significant right upper quadrant tenderness. Review of systems was negative for fever, weight loss, night sweats, respiratory symptoms, and cardiac symptoms. Neurological exam revealed no focal deficits.

A CT scan of the abdomen and pelvis revealed extensive periportal, upper abdominal, and periaortic lymphadenopathy, along with a 2.8 cm left adnexal cyst, raising concern for a metastatic process. These findings prompted a comprehensive serologic work up for infection and malignancy.

With the concern for a metastatic process, a brain MRI was performed for staging. It showed a punctate, faint enhancement of the right medial occipital lobe, which was thought to be vascular in nature. No evidence of intracranial metastatic disease was found.

The patient was found to have reactive HIV antibodies, with a CD4 count of 145 cells/uL and a viral load of 158,000 copies/mL. She tested positive for syphilis total IgG and IgM with a negative RPR. Toxoplasma gondii antibodies were positive, while testing for *Neisseria gonorrhoeae* and *Chlamydia trachomatis* were negative. Serum CrAg LFA was negative at this time.

A CT scan of the chest revealed diffuse, millimetric nodularity of the lungs in a random pattern and extensive lymphadenopathy, raising suspicion for metastatic disease, infection, or disseminated tuberculosis. Although the patient denied pulmonary symptoms, she had close contact with an individual diagnosed with active tuberculosis. Sputum acid-fast bacilli (AFB) cultures were positive for *Mycobacterium tuberculosis* (TB) complex. A lymph node biopsy confirmed the diagnosis of active miliary TB.

Infectious disease specialists were consulted, and the patient was started on RIPE therapy (rifampin, isoniazid, pyrazinamide, and ethambutol) for treatment of TB. She ultimately was discharged on RIPE therapy with plans to follow up with infectious disease specialists to determine the optimal timing for initiating antiretroviral therapy for her new diagnosis of HIV.

Unfortunately, the patient returned 8 days later with difficulty tolerating oral intake, new frontal headaches, orbital pain, new visual hallucinations, and photophobia without neck pain or nuchal rigidity. She was febrile, tachycardic, and ill-appearing. She reported adherence to her RIPE therapy since discharge. Infectious disease specialists were consulted and initially suspected this was an adverse reaction to isoniazid. However, a CT scan of the head revealed subtle hypoattenuation of the right medial occipital lobe, corresponding to prior MRI findings, now concerning for developing sequela of an atypical or opportunistic infection given the patient's recent HIV diagnosis. A repeat brain MRI showed similar nonspecific curvilinear leptomeningeal or vascular enhancement of the right medial occipital lobe, thought to be vascular in nature, similar to the initial MRI during the earlier admission. A tiny 3 mm dural-based lesion along the right anterior falx was noted, likely representing a meningioma. Mild supratentorial ventriculomegaly was also observed.

Given her infectious presentation, new neurological symptoms, and active HIV and TB infections, a broad infectious workup was conducted. Lumbar puncture was performed, and cerebrospinal fluid studies were positive for CrAg, with CSF gram stain showing moderate yeast. The diagnosis of cryptococcal meningitis was made and she was started on amphotericin B and flucytosine.

After CSF cultures confirmed cryptococcal meningitis, a repeat serum CrAg LFA was obtained, which was positive with a titer of exceeding 1:2560. Blood cultures grew *Cryptococcus neoformans*, revealing a further diagnosis of disseminated cryptococcosis.

The patient was started on induction therapy with a 14-day course of amphotericin B and flucytosine. She continued to have persistent headaches and vision changes throughout her admission. Two additional lumbar punctures were performed due to elevated intracranial pressure and persistent headaches. The CSF remained positive for CrAg. At discharge, her headache and vision changes had resolved. After completion of induction therapy with amphotericin and flucytosine, she was transitioned to fluconazole for continued treatment of disseminated cryptococcosis.

## 3. Discussion

This case emphasizes the importance of cerebrospinal fluid testing for *Cryptococcus neoformans* in high-risk patients, even if initial serum CrAg results are negative. Although the serum CrAg LFA boasts remarkable sensitivity and specificity, false negatives can occur, delaying diagnosis and treatment initiation. This is a rare case where serum CrAg, brain MRI, and symptoms were not suggestive of cryptococcal disease despite the patient likely having active cryptococcal infection. Clinicians should remain vigilant and consider performing a lumbar puncture with CSF sampling when there is a high suspicion for fungal infection.

A positive serum CrAg typically precedes the onset of meningitis symptoms by several weeks. In this case, the suspicion for cryptococcal meningitis was low due to the negative serum CrAg screening that was performed 8 days before the development of concerning neurologic symptoms. At this point in time, CSF testing was performed for evaluation of other opportunistic infections.

Brain imaging, particularly MRI, can aid in diagnosis of cryptococcal meningitis. Common MRI findings in cryptococcal infection include dilated perivascular spaces and basal ganglia pseudocysts. However, in severely immunosuppressed patients, leptomeningeal enhancement may be less reliable because these patients often cannot mount an adequate immune response necessary to produce such findings [7]. This limits the reliability of MRI findings in immunocompromised patients.

This case raises awareness that a negative serum CrAg and absent brain MRI findings are not sufficient to rule out cryptococcal infection in immunocompromised individuals. When the index of suspicion for cryptococcal infection is high, clinicians should proceed with lumbar puncture and CSF testing to ensure accurate diagnosis and timely treatment.

## Data Availability

All data generalized or analyzed during this study are included within the article.
